# Gα_12_ and Gα_13_: Versatility in Physiology and Pathology

**DOI:** 10.3389/fcell.2022.809425

**Published:** 2022-02-14

**Authors:** Paipai Guo, Yu Tai, Manman Wang, Hanfei Sun, Lingling Zhang, Wei Wei, Yang K. Xiang, Qingtong Wang

**Affiliations:** ^1^ Key Laboratory of Anti-inflammatory and Immune Medicine, Ministry of Education, Collaborative Innovation Center of Anti-inflammatory and Immune Medicine, Institute of Clinical Pharmacology, Anhui Medical University, Hefei, China; ^2^ Department of Pharmacology, University of California, Davis, Davis, CA, United States; ^3^ VA Northern California Health Care System, Mather, CA, United States

**Keywords:** G protein-coupled receptor, Gα_12_, Gα_13_, cell pathophysiology, diseases

## Abstract

G protein-coupled receptors (GPCRs), as the largest family of receptors in the human body, are involved in the pathological mechanisms of many diseases. Heterotrimeric G proteins represent the main molecular switch and receive cell surface signals from activated GPCRs. Growing evidence suggests that Gα_12_ subfamily (Gα_12/13_)-mediated signaling plays a crucial role in cellular function and various pathological processes. The current research on the physiological and pathological function of Gα_12/13_ is constantly expanding, Changes in the expression levels of Gα_12/13_ have been found in a wide range of human diseases. However, the mechanistic research on Gα_12/13_ is scattered. This review briefly describes the structural sequences of the Gα_12/13_ isoforms and introduces the coupling of GPCRs and non-GPCRs to Gα_12/13_. The effects of Gα_12/13_ on RhoA and other signaling pathways and their roles in cell proliferation, migration, and immune cell function, are discussed. Finally, we focus on the pathological impacts of Gα_12/13_ in cancer, inflammation, metabolic diseases, fibrotic diseases, and circulatory disorders are brought to focus.

## Introduction

G protein-coupled receptors (GPCRs) family are a superfamily of membrane receptors responsible for signal transduction in cells. GPCRs are extensively studied drug targets because they participate in a broad range of human physiological and pathological processes. There are currently 481 drugs (about 34% of all drugs approved by the FDA) acting on 107 unique GPCRs to treat different diseases, including neurological disorders, metabolic and cardiovascular diseases, cancer, and inflammation (Hauser et al., 2017; Wang et al., 2020). Heterotrimeric G proteins are sensors for GPCR active conformations and trigger intracellular signal transduction (Maziarz et al., 2020). Heterotrimeric G proteins are composed of Gα, Gβ, and Gγ subunits, which are mainly located on the inner leaflet of the plasma membrane (Bondar and Lazar, 2021). Gα proteins are divided into four categories based on sequence homology and downstream effectors: Gα_s_ (s stands for stimulation), Gα_i/o_ (i stands for inhibition), Gα_q/11_, and Gα_12/13_ (Hilger et al., 2018; Yang et al., 2020). The function of Gα_s_, Gα_i/o_, and Gα_q_ have been well documented. Meanwhile, the progress in understanding the function of the Gα_12/13_ family, which was discovered in the early 1990s, has been relatively slow (Kim et al., 2018a). Nevertheless, with the development of new research tools (for example, constitutively active mutants, fusion proteins, and gene knockout, etc.), progress has been made in further to understanding the function of Gα_12/13_ in recent years (Worzfeld et al., 2008).

Gα_12/13_ subunits are expressed in most cell types and are able to induce diversified cellular signaling and responses that are important players in health and disease. Gα_12_ and Gα_13_ share 67% of their amino acid sequence and have many downstream signaling targets in commm (Montgomery et al., 2014; Stecky et al., 2020). Some of the pathways that are triggered by both Gα_12_ and Gα_13_ include phospholipase C (PLC)-ε and phospholipase D, mitogen-activated protein kinase (MAPK), and Na/H-exchange which promote cytoskeletal alterations, carcinogenic responses, and apoptosis (Litosch, 2012; Dusaban et al., 2013; Xie et al., 2016). Moreover, Gα_12/13_ interacts with specific guanine nucleotide exchange factors (GEFs) (e.g., p115RhoGEF, leukemia-related RhoGEF, and PDZ-RhoGEF) to activate downstream effectors, including ras homolog family member A (RhoA), PLC, adenylate cyclase, and a variety of ion channels (Mikelis et al., 2013). These effectors, in turn, regulate the concentration of secondary messengers in the cells, such as diglycerides, cyclic adenosine monophosphate (cAMP), sodium ions, and calcium ions. Together, the activated RhoA and sencodary messengers eventually lead to physiological responses (Jiang et al., 2008; Kalwa et al., 2015; Wang et al., 2019). Furthermore, Gα_12/13_ subunits also regulate the activity of a variety of transcription factors, such as signal transducer and activator of transcription 3, serum response factor (SRF), activator protein 1 (AP-1), and activated T cell nuclear factor (NFAT) (Kumar et al., 2006; Lee et al., 2009; Song et al., 2018; Yagi et al., 2019). Abnormally elevated upstream stimuli promote the incidence and development of diseases by increasing the corresponding receptor coupling to Gα_12_ and/or Gα_13_ (Yang et al., 2020; Rasheed et al., 2021). In this review, the structure of Gα_12_ and Gα_13_ is reviewed along with their roles in GPCR signal pathways, cell function, and disease pathogenesis.

### The Amino Acid Structure of Gα_12_ and Gα_13_

The Gα_12/13_ subfamily consists of two *α* subunits encoded by *GNA12* and *GNA13*. The Gα_12/13_ subunits were originally discovered based on the amino acid sequence similarity with other Gα subunits and their insensitivity to pertussis toxin (Arang and Gutkind, 2020). The structure of Gα_12/13_ subunit consists of an amino-terminal *α*-helical domain and a Ras-like GTPase domain. There is a link between these two domains involved in binding to GDP and GTP (Syrovatkina et al., 2016; Smrcka and Fisher, 2019). In response to activation of GPCR, the conformation of GDP-bound inactive Gα_12/13_ is transformed into the active form with GTP binding, triggering the dissociation of Gα_12/13_ from Gβγ and activation of downstream effectors (Arthofer et al., 2016). After dissociation, free Gβγ subunits transmit signals through regulating canonical effectors, including adenylate cyclase, PLC, and various ion channels (Senarath et al., 2018). Gβγ subunits also regulate a series of non-canonical effectors, such as the nuclear import of the extracellular regulated protein kinases (ERK) 1/2, oxidative phosphorylation, and mRNA processing (Khan et al., 2016). The large number of Gβγ subunits in mammal cells define much of the diversity that occurs within GPCR signaling with resepect to spatial and temporal bias and are extensively involved in the pathogenesis of diseases (Masuho et al., 2021). Gβγ signaling has been previously well summarized.

**FIGURE 1:**
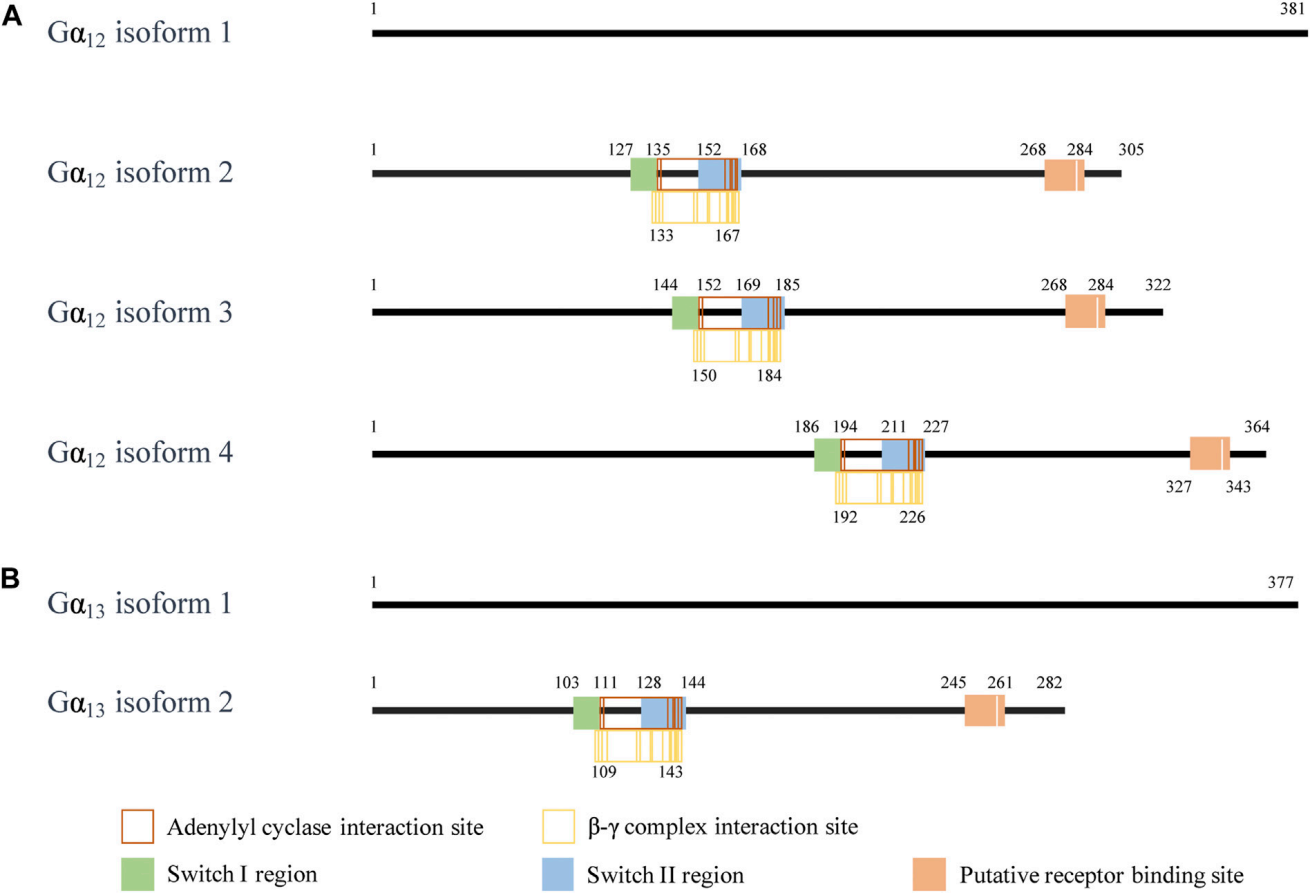
The interaction site of the Gα_12/13_ isoform. **(A)** The interaction sites on the amino acid fragment of Gα_12_ isoforms. **(B)** The interaction sites on the amino acid fragment of Gα_13_ isoforms. The information on the interaction sites of Gα_12_ and Gα_13_ is from NCBI. In NCBI reference sequence database, the human *GNA12* isoforms are NP_031379.2, NP_001269369.1, NP_001269370.1, and NP_001280021.1. (https://www.ncbi.nlm.nih.gov/gene/2768). The human *GNA13* isoforms are NP_006563.2 and NP_001269354.1. (https://www.ncbi.nlm.nih.gov/gene/10672).

Although Gβγ-mediated effects of GPCR signaling are diverse, the assorted isoforms of Gα also have a wide variety of influences. These varied functions are highly related to their structures. Under present consideration, Gα_12_ has four isoforms, of which isoform 1 is the longest isoform containing 381 amino acids (Figure 1). Compared with isoform 1, the encoded isoform 2 (305 amino acids) and isoform 3 (322 amino acids) are shorter and have different N-termini. Both Gα_12_ isoform 2 and 3 have distinct 5′ untranslated region and 5′ coding region for different N-termini. The isoform 4 of Gα_12_ lacks in-frame exons in the 3′ coding region relative to the isoform 1; the encoded isoform 4 (364 amino acids) is also shorter than isoform 1. Gα_13_ has two isoforms, of which isoform 1 is the longer isoform containing 377 amino acids. The isoform 2 of Gα_13_ uses an alternative 5′ exons resulting in a downstream start codon AUG. Thus, the encoded isoform 2 of Gα_13_ has a shorter N-terminus than the isoform 1. The isoform sequences of Gα_12_ or Gα_13_ show similar structural domains, including an adenylyl cyclase binding site, a β-γ complex binding site, a switch I region (one of two surface loops that undergo conformational changes upon GTP binding), a switch II region, and a putative receptor binding site, respectively, (Lambright et al., 1996; Sunahara et al., 1997; Tesmer et al., 1997). Additional difference in the amino acid sequence of each isoform may provide tissue specific expression and cellular localization to fulfill the broad functional roles of the Gα_12_ or Gα_13_.

While literature clearly show that Gα_12/13_ has different functions from other Gα subtypes, the functional difference between the isoforms of Gα_12_ and Gα_13_ has not been reported. Future studies on the structure-functional relationship between these isoforms will help understanding their roles in cells and diseases.

### Regulation Network of Gα_12/13_

Gα_12/13_ has been shown to couple to more than 30 GPCRs. Activated by various upstream stimuli, Gα_12/13_ can transmit to divergent downstream signaling pathways and regulates cell function in pathophysiological processes (Ackerman et al., 2015; Yung et al., 2017; Yanagida et al., 2018; Spoerri et al., 2020). Gα_12_ and Gα_13_ are broadly expressed, yet gene deficiency in mice shows that they are not interchangeable (Yang et al., 2020). Some GPCRs preferentially couple to either Gα_12_ or Gα_13_ (Ayoub and Pin, 2013; Yung et al., 2017; Mackenzie et al., 2019). Moreover, non-GPCRs are also shown to couple to Gα_12_ and Gα_13_ (Shen et al., 2013; Shen et al., 2015; Piccinin et al., 2019) (Table 1).

**TABLE 1:** List of biologically significant Gα_12/13_-associated receptors and their physiological functions.

Receptors	G proteins	Functions	References
		GPCRs	
LPA receptor	Gα_12_	Migration of fibroblasts	Gan et al. (2013)
LPA receptor	Gα_12/13_	Embryonic blood vessel development	Tanaka et al. (2006)
LPAR1	Gα_12_	Proliferation of astrocytes	Loskutov et al. (2018)
LPAR1/LPAR2	Gα_12/13_	Cell proliferation, migration, and cytoskeleton changes	Kihara et al. (2014)
LPAR4/LPAR6	Gα_12/13_	Changes in the tension of the actin cytoskeleton	(Noguchi et al. (2003), Yanagida et al. (2009)
LPAR4/LPAR6	Gα_12/13_	Angiogenesis	Yasuda et al. (2019)
LPAR5	Gα_12/13_	Axon retraction and stress fiber formation	Lee et al. (2006)
FZD	Gα_12/13_	Bone formation and cell migration	Park et al. (2015)
FZD	Gα_12/13_	Bone homeostasis	Hendrickx et al. (2020)
FZD4	Gα_12/13_	Angiogenesis	Arthofer et al. (2016)
FZD10	Gα_13_	Angiogenesis	Hot et al. (2017)
PAR1/PAR2	Gα_13_	Fibroblast adhesion maturation, spreading, and migration	Spoerri et al. (2020)
PAR1	Gα_12/13_	Stress fiber formation	Regué et al. (2013)
PAR2	Gα_13_	Smooth muscle contraction	Sriwai et al. (2013)
S1PR1/S1PR3/S1PR5	Gα_12_	Inflammation	Ki et al. (2007)
S1PR2	Gα_12/13_	Vascular smooth muscle cell migration and neointimal hyperplasia	Kim et al. (2011)
S1PR2	Gα_12/13_	Myofibroblast contraction	Sobel et al. (2015)
S1PR2	Gα_13_	Cardiomyocyte migration	Ye and Lin, (2013)
S1PR2/S1PR3	Gα_12/13_	Stress fiber formation	Olivera et al. (2003)
S1PR2/S1PR3	Gα_12/13_	Cardiac progenitor cell proliferation	Castaldi et al. (2016)
S1PR3	Gα_12/13_	Inflammation	Dusaban et al. (2017)
S1PR3	Gα_13_	Cardioprotection	Yung et al. (2017)
M1R	Gα_13_	Impaired growth	Sabbir et al. (2018)
M3R	Gα_12_	Human airway smooth muscle cells contraction	Yoo et al. (2017)
CXCR4	Gα_13_	Tumor cell migration and adhesion	Scarlett et al. (2018)
CXCR4	Gα_13_	Breast cancer metastasis	Yagi et al. (2011)
Calcium-sensing receptor	Gα_12/13_	Gene expression, cytoskeleton, and cell shape	Leach et al. (2012), Leach et al. (2013)
AT_1_R	Gα_12/13_	Vasoconstriction	Lymperopoulos et al. (2021)
Thromboxane A2 receptor	Gα_12/13_	Neointima formation and restenosis	Feng et al. (2016)
Ghrelin receptor	Gα_12_	Food intake	Mende et al. (2018)
Dopamine D3 receptor	Gα_12_	Inhibit inflammation	Wang et al. (2015)
5-HT_4_R	Gα_13_	Hippocampal synaptic function	Müller et al. (2021)
5-HT_4_R	Gα_13_	Angiogenesis	Profirovic et al. (2013)
UII receptor	Gα_13_	Tumor invasion	Lecointre et al. (2015)
Complement C5a receptor	Gα_12/13_	Macrophage tail retraction	Raghavan et al. (2018)
GPR56	Gα_12/13_	Myelination	Ackerman et al. (2015)
Purinergic receptor 6	Gα_13_	Cell migration	Girard et al. (2020)
Gastrin type 2 cholecystokinin receptor	Gα_13_	Cell migration	Masià-Balagué et al. (2015)
GPR40	Gα_12/13_	Release of insulin vesicles	Rives et al. (2018)
GPR56	Gα_12/13_	Muscle protein synthesis and myotube hypertrophy	White et al. (2014)
GPR91	Gα_12_	Mitochondrial fission and cell migration	Ko et al. (2017)
ET-1 type A receptor	Gα_12/13_	Formation of myofibroblasts	Nishida et al. (2007)
		**Non-GPCRs**	
Integrin β_1_	Gα_13_	Cell migration	Shen et al. (2013), Shen et al. (2015)
Integrin α_IIb_β_3_	Gα_13_	Cell retraction and migration	Gong et al. (2010)
Integrin α_IIb_β_3_	Gα_13_	Promote thrombosis	Pang et al. (2018)
PPARγ	Gα_13_	Inhibit thrombosis	Unsworth et al. (2017)
Smoothened	Gα_12_	Tumor growth and anti-apoptosis	Qu et al. (2013)

### GPCRs Triggered Gα_12/13_ Signaling

#### Lysophosphatidic Acid Receptors

Lysophosphatidic acid (LPA) is a biologically active phospholipid, which mediates various biological functions through six homologous LPA receptors (LAPRs) (Plastira et al., 2020). Activated LPARs promote Gα_12_, but not Gα_13_, association with v-raf murine sarcoma viral oncogene homolog A which than activates ERK. The activated ERK promotes ring finger and FYVE like domain-containing E3 ubiquitin protein ligase transcription and fibroblast migration (Gan et al., 2013). LPAR-Gα_12/13_ signaling also plays an important role in embryonic blood vessel development (Tanaka et al., 2006). Additionally, LPAR1/2 couple with Gα_i/o_, Gα_q/11_, as well as Gα_12/13_ to activate a variety of downstream pathways, such as protein kinase B (PKB, commonly known as AKT), RhoA, MAPK, and phosphatidylinositol 3-kinase (PI3K), and regulates cell proliferation, migration, and cytoskeleton rearrangement (Kihara et al., 2014). Previous studies have identified that LPAR4/6 can effectively couple to Gα_12/13_ to stimulate Rho GTPase, which subsequently activates Rho-associated protein kinase (ROCK) I/II, leading to changes in the tension of the actin cytoskeleton (Noguchi et al., 2003; Yanagida et al., 2009). The LPAR4/6-activated Gα_12/13-_Rho-ROCK signal also promotes nuclear translocation of Yes-associated protein/transcriptional coactivator with PDZ-binding motif (YAP/TAZ) (Yasuda et al., 2019). YAP/TAZ promotes the proliferation of cancer cells (such as liver, bladder, and lung cancers) and accelerates the progress of cancer (Yagi et al., 2016; Maziarz et al., 2020). Activation of LPAR5 also induces axon retraction and stress fiber formation through an LPA-LPAR5-Gα_12/13_ pathway (Lee et al., 2006). As of today, there is no evidence supporting a coupling between LPAR3 and Gα_12/13_.

### Frizzleds

Frizzled (FZD) receptors are unconventional GPCRs, which can be activated by the *Wingless/Int-1* lipoglycoprotein (WNT) family (Janda et al., 2017). FZD4 interacts with Gα_12/13_ and does not interact with other subunits of the G protein family. The complex formed by FZD4 and Gα_12/13_ is dissociated under WNT stimulation. The FZD4-Gα_12/13_ signaling mediates cytoskeletal rearrangement and Rho signaling through p115RhoGEF, affecting angiogenesis in embryonic and tumor development (Arthofer et al., 2016). WNT5a/b and WNT3a bind to the receptor tyrosine kinase-like orphan receptor 1/2-FZD complex, activating Rho GTPases through Gα_12/13_. The activated Rho inhibits the activity of large tumor suppressor 1/2 (LATS1/2), leading to YAP/TAZ dephosphorylation and nuclear translocation and promoting bone formation and cell migration (Park et al., 2015). Interestingly, a similar mechanism has been found in brain endothelial cells. The FZD10-Gα_13_ complex dissociates under WNT5a/7a stimulation, and Gα_13_ transmits a signal to YAP/TAZ through Rho family members (Hot et al., 2017). In osteoblasts, WNT family member 16 binds to FZD receptors to activate canonical WNT signaling and non-canonical Gα_12/13_ signaling and regulate bone homeostasis (Hendrickx et al., 2020).

### Protease-Activated Receptors

Protease-activated receptors (PARs) consist of four subtypes (PAR1-4). PARs play an important role in blood vessel development, cell proliferation, tumorigenesis, and thrombosis (Chandrabalan and Ramachandran, 2021). In fibroblasts, PAR1/2 send signals to integrin α_5_β_1_ through Gβγ and PI3K to induce fibronectin binding and initiate cell adhesion. PAR1/2 also send signals through Gα_13_, Gα_i_, ROCK, and Src to enhance integrin α_5_β_1_-mediated adhesion (Spoerri et al., 2020). The PAR1-mediated Gα_12/13_ activation stimulates RhoGEF and simultaneously activates the RhoA-ROCK pathway and myosin light chain (Flaumenhaft and De Ceunynck, 2017). Stimulation of PAR1 also initiates the assembly of F-actin through the Gα_12/13_-RhoA pathway, which induces YAP dephosphorylation and nuclear translocation, thereby promoting cell migration and invasion (Regué et al., 2013). PAR2 couples with Gα_q_, Gα_i_, and Gα_13_ to stimulate RhoA-ROCK activity independently of the cyclic adenosine monophosphate-protein kinase A pathway to induce smooth muscle contraction (Sriwai et al., 2013). An earlier study found that in endothelial cells, PAR3 directly interacts with PAR1 to change the binding conformation of PAR1/Gα_13_, induce the activation of downstream signaling pathways, and promote endothelial barrier dysfunction (McLaughlin et al., 2007). Presently, there is no clear evidence showing a direct interaction between PAR3/4 and Gα_12/13_.

### Sphingosine 1-Phosphate Receptors

Sphingosine 1-phosphate (S1P) is a natural biologically active lipid molecule that binds to five different S1P receptors (S1PR1-5). Activated S1PRs couple with Gα_12/13_ to induce downstream signals (Zhang et al., 2020). An earlier study shows that S1PR2/S1PR3 couple to Gα_12/13_ and send an “inside-out” signal to mediate the formation of stress fibers (Olivera et al., 2003). After binding to S1P, S1PR1/3/5 couple with Gα_12_ and activate the c-Jun N-terminal kinase (JNK)-nuclear factor-kappa B (NF-κB) pathway to promote the expression of cyclooxygenase-2 and accelerate local inflammation (Ki et al., 2007). S1PR3 activates the Gα_12/13_-RhoA pathway and promotes the expression of inflammatory gene in astrocytes (Dusaban et al., 2017). S1PR3 activates Gα_13_-RhoA in cardiomyocytes and mediates cardio-protection during ischemia/reperfusion (I/R) (Yung et al., 2017). In vascular smooth muscle cells, S1PR2 couples to Gα_12/13_ to activate AP-1-dependent induction of cysteine-rich protein 61 and promote the migration of vascular smooth muscle cells and neointimal hyperplasia (Kim et al., 2011). S1PR2 also induces the contraction of myofibroblasts through the Gα_12/13_-Rho-ROCK pathway (Sobel et al., 2015). Moreover, Gα_12_, activated by S1PR2, is recruited to E-cadherin to form a complex after mechanical stress. Gα_12_ then recruits and activates p114RhoGEF, driving RhoA signaling and increasing the tensile strength of multicellular connections (Acharya et al., 2018).

### Muscarinic Acetylcholine Receptors

Muscarinic acetylcholine receptors have five different subtypes (M1R-M5R) (Ruan et al., 2021). The increase of acetylcholine signal in neurons leads to the overexpression of M1R, promoting the polymerization of Gα_13_ and Gβγ (Sabbir et al., 2018). Gα_13_ disrupts the stability of tubulin polymer through the RhoA-ROCK signal, reducing mitochondrial transport and impairing growth in neurites (Sabbir et al., 2018). Early study has shown that M3R promotes the activity of phospholipase D through Gα_12_ in HEK-293 cells (Rümenapp et al., 2001). It has also been found that Gα_12_ binds to M3R in human airway smooth muscle cells. The M3R-Gα_12_ signaling is important in promoting the contraction of human airway smooth muscle cells by inducing PI3K-mediated ROCK activation in a RhoA-dependent manner (Yoo et al., 2017).

### Chemokine Receptors

The binding of chemokine C-X-C motif chemokine 12 (CXCL12) to C-X-C chemokine receptor 4 (CXCR4) activates RhoA through Gα_13_, which leads to ROCK phosphorylation of myosin light chain and promotes cell migration and adhesion (Scarlett et al., 2018). In metastatic breast cancer cells, CXCR4 activates small Rho GTPases through Gα_13_ to initiate cell motility and trans-endothelial migration (Yagi et al., 2011).

### Non-GPCRs Triggered Gα_12/13_ Signaling

Integrin “outside-in” signaling requires Gα_13_ and monomeric small G proteins (i.e., Rho) to mediate cell retraction, migration, and spreading (Shen et al., 2012). Gα_13_ directly binds to the ExE motif in the cytoplasmic domain of the integrin β_3_ subunits, which is important in transducing the “outside-in” integrin signaling (Shen et al., 2013). Gα_13_ also binds to the cytoplasmic domain of the integrin β_1_ subunit in platelets, which mediates the Src-dependent transient inhibition of RhoA, activates the Rac1 and PI3K pathways, and promotes cell migration (Shen et al., 2013; Shen et al., 2015). Interfering with the expression of Gα_13_ reduces α_IIb_β_3_-dependent activation of c-Src, inhibits cell migration, and accelerates cell contraction, thereby spreading platelets on fibrinogen (Gong et al., 2010). Integrin α_IIb_β_3_ also serves as a mechanical sensor that transmits “outside-in” signals through Gα_13_-Src-Rac1-dependent pathways in platelets and facilitates coagulation *in vitro* and intravascularly *in vivo* (Pang et al., 2018). Therefore, the Gα_13_-integrin interaction is important in thrombosis.

Additionally, peroxisome proliferator-activated receptor-γ (PPARγ), a member of the nuclear hormone superfamily, regulates lipid and glucose metabolism and homeostasis in many metabolic pathways (Piccinin et al., 2019). Treatment of platelets with PPARγ agonists leads to decreased binding between Gα_13_ and integrin β_3_, which prevents c-Src-dependent integrin β_3_ phosphorylation and talin dissociation and weakens the downstream signal transduction of integrin α_IIb_β_3_, thereby regulating platelet activation and reducing thrombosis (Unsworth et al., 2017). Moreover, in diffuse large B-cell lymphoma (DLBCL), smoothened recruits Gα_i_ and Gα_12_ and activates the protein kinase C (PKC)-caspase recruitment domain and membrane-associated guanylate kinase-like domain protein 1-dependent signaling cascade, which promotes activation of NF-κB, tumor growth, and anti-apoptosis (Qu et al., 2013).

### Gα_12/13_ and Biased Signaling

Traditionally, each GPCR is thought to initiate the “canonical” signal transduction through a single homologous G protein class (Seyedabadi et al., 2019). With the advances in the study of the ligand-receptor-effector relationship, it has been found that specific ligands induce a GPCR to selectively bind to a particular G protein subunit, transducing biased intracellular signaling toward one of many downstream pathways. This phenomenon is called “biased signaling”; and functionally selective ligands are called “biased ligands” (Tan et al., 2018).

Up to now, only a few GPCRs have shown biased signaling, but it is still the early days of understanding the biased signaling mechanisms. The characteristics of biased ligands, which have strong or weak activity in different pathways, may provide significant clinical advantages for developing new drugs. However, attention should be paid to testing conditions, ligand verification, and patient and disease selection to achieve successful biased ligand therapy (Seyedabadi et al., 2019). So far a few of the receptor types seen to produce biased signaling involving Gα subunit switch include calcium-sensing receptors, angiotensin receptors, PARs, prostaglandin receptors, and ghrelin receptor.

The biased signaling can occur due to different underlying principles. The well-documented biased signaling is triggered by biased ligands. For instance, calcium-sensing receptor induces the activation of four G protein subfamilies (Gα_q/11_, Gα_i/o_, Gα_12/13_, and Gα_s_) (Abid et al., 2021). The ligands NPS-2143/NPS-R568 binds to a calcium-sensing receptor to specifically mediate the activation of Gα_12/13_-RhoA signal, which in turn activates various other signal checkpoints that regulate gene expression, cytoskeleton, and cell shape (Leach et al., 2012; Leach et al., 2013). Similarly, the prostaglandin F2α receptor, a Gα_q_-coupled GPCR, is activated by prostaglandin F2α (Pathe-neuschäfer-rube et al., 2005). PDC113.824 acts on the prostaglandin F2α receptor, which biasedly increases Gα_q_-PKC-ERK1/2 signaling while inhibiting Gα_12_-Rho-ROCK signaling, blocking cell contraction and skeletal reorganization, and inhibiting uterine contraction (Goupil et al., 2010). PAR2 is activated mainly by trypsin-like serine proteases and regulates various signaling pathways in coupling with Gα_i/o_, Gα_q/11_, and Gα_12/13_ (Kim et al., 2018b). Among PAR2 antagonists, I-287 inhibits Gα_q_, but biasedly activates Gα_12/13_ without affecting Gα_i/o_ signaling and β-arrestin recruitment, thereby attenuating the PAR2-mediated inflammatory response (Avet et al., 2020). Likewise, the ghrelin receptor in the arcuate nucleus of the hypothalamus, activated by Ghrelin, induces an intracellular signaling cascade through Gα_q_, Gα_i/o_, Gα_12/13_, and β-arrestin (Hedegaard and Holst, 2020). The biased ligand YIL781 selectively activates Gα_q/11_ and Gα_12_ through the ghrelin receptor without intrinsic activity for β-arrestin recruitment, leading to an increased food intake and a reduced gastric emptying (Mende et al., 2018). However, other factors, including ionic strength, lipid environments, and downstream signaling partners, can also contribute to biased signaling observed in native cells and tissues. For instance, receptors can couple in a cell type-specific manner. The binding of angiotensin II (Ang II) to the Ang II type 1 receptor (AT_1_R) causes AT_1_R interaction with Gα_q/11_, Gα_12/13_, and Gα_i_, depending on cell type (Forrester et al., 2018). When Ang II stimulates vascular smooth muscle cells, AT_1_R binds to Gα_12/13_ instead of Gα_q/11_ to activate RhoA-ROCK and promote vasoconstriction (Lymperopoulos et al., 2021). Cleaving a receptor is another method that often leads to a change in downstream signaling. For PAR1, the endogenous ligand thrombin promotes PAR1 binding to heterotrimeric G proteins of the Gα_q/11_, Gα_12/13_, Gα_i_, and Gα_s_ families. The activation of matrix metalloproteinase-1 in platelets cleaves the N-terminal extracellular domain of PAR1, activating the Gα_12/13_-Rho-MAPK signal instead of Gα_q/11_, and promotes cell shape changes and platelet thrombosis (Trivedi et al., 2009).

### Gα_12/13_Signaling and Cell Function

**FIGURE 2:**
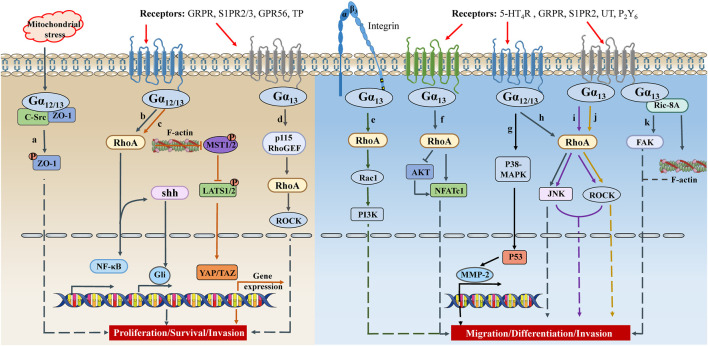
The crosstalk between Gα_12/13_ signaling and cell proliferation/migration. Under different physiological/pathological environments, GPCRs on the cell membrane receive different signals, couple Gα_12_ or Gα_13_, activate signal cascades, and promote synthesis of transcription factors or secretion of inflammatory factors. These signals ultimately lead to different cell activities, such as cell proliferation, survival, differentiation, migration, invasion, etc. TP, thromboxane A2 receptor; UT, urotensin II receptor; P_2_Y_6_, purinergic receptor 6; shh, sonic hedgehog; MST1/2, mammalian sterile 20-like kinases 1/2.

As discussed above, the well-characterized downstream effector of active Gα_12/13_ is the Rho GTPases through stimulating RhoGEF, which regulates the actin cytoskeleton and participates in various cellular functions, including cell proliferation, migration, contractility, and gene expression (Bodmann et al., 2017) (Figure 2).

### Cell Growth and Apoptosis

Gα_12/13_ was initially identified as an oncogene with the potential for tumor transformation of fibroblasts (Chan et al., 1993; Xu et al., 1993). Subsequent studies have shown that Gα_12/13_ can promote mitogenic response and cell growth by transducing a RhoA-dependent signal, which increases YAP/TAZ-dependent gene expression (Goldsmith and Dhanasekaran, 2007; Syrovatkina and Huang, 2019).

Subsequent publications have revealed that Gα_12/13_ coupling with several different receptor types promotes proliferation. S1P activates S1PR2/3 to trigger Gα_12/13_-RhoA signaling, leading to the proliferation of mouse cardiac progenitor cells and regulating gene transcription in hearts (Castaldi et al., 2016). Stimulation of the thromboxane A2 receptor also promotes the activity of Rho GTPase through Gα_12/13_. The activated Rho GTPase regulates actin cytoskeleton, increases nuclear translocation of YAP/TAZ, and promotes proliferation and migration of T/G HA-vascular smooth muscle cells (Feng et al., 2016). After the loss of primary cilia on human astrocytes, LPA promotes association between LPAR1 and Gα_12_/Gα_q_, augmenting mitogenic signaling and cell proliferation (Loskutov et al., 2018). Furthermore, the acetylcholine signaling via M1R activates Gα_13_ protein to disrupt tubulin polymerization in axons and inhibit mitochondrial transport, thereby limiting the growth of neurites (Sabbir et al., 2018). G protein-coupled receptor 56 (GPR56) interacts with Gα_12/13_ to mediate RhoA signaling and regulates zebrafish oligodendrocytes’ development and subsequent myelination (Ackerman et al., 2015).

Gα_12/13_ is also very important in the proliferation of tumor cells. The activation of Gα_12/13_ promotes cell growth and tumor development of hepatocellular, small cell lung carcinoma, and ovarian cancer cells, but not in breast and prostate cancer cells (Grzelinski et al., 2010; Rasheed et al., 2013; Yagi et al., 2016; Syrovatkina and Huang, 2019). The synthetic ligand, Clozapine N-oxide, stimulates GPCR-Gα_12/13_ signaling and promotes the proliferation of ovarian cancer cells by activating YAP1 (Yagi et al., 2016). Bombesin secreted by small cell lung carcinoma cells activates the gastrin-releasing peptide receptor (GRPR)-Gα_12/13_-Rho-NF-κB signaling cascade. Subsequently, the activated NF-κB increases the production of Sonic Hedgehog, which activates the Gli transcription factor and promotes cell proliferation, survival, blood vessel generation, and local invasion (Castellone et al., 2015).

Meanwhile, the combination of muscle-restricted coiled-coil protein and caveolin-1 promotes Gα_13_-mediated p115RhoGEF activation, leading to subsequent activation of the Rho-ROCK signal and enhancing the proliferation and migration of human pulmonary artery smooth muscle cells (Nakanishi et al., 2016). Moreover, Gα_13_ dynamically regulates the RhoA signaling through the combination of RhoGEF GTPase and integrin β_1_, promoting integrin β_1_-mediated proliferation of CHO cell lines (Shen et al., 2015). In addition to autophagy-mediated mitochondrial damage and oxidative stress, lysosomal dysfunction in cystopathy stimulates Gα_12_/Src-mediated phosphorylation of zona occludin-1. The activated zona occludin-1-related signaling cascade promotes the proliferation of epithelial cells and disrupts the cell lining along the proximal tubules of mouse kidneys (Festa et al., 2018).

Studies have also found that Gα_12_ and Gα_13_ regulate apoptosis. In Madin-Darby canine kidney cells, the activation of endogenous Gα_12_ and thrombin stimulation increase the activity of JNK1, inhibit the activity of NF-κB, and promote cell apoptosis (Yanamadala et al., 2007). In melanoma cells, US28, a GPCR encoded by human cytomegalovirus, is coupled with Gα_13_ to induce cell apoptosis; silencing Gα_13_ inhibits cell apoptosis driven by US28 (Joshi et al., 2015). Notably, this event has been only observed in human melanoma cell lines but not in murine cells. The emerging discovery of ligand-GPCR-Gα_12/13_ signals will offer insight into the regulation of apoptosis on cancer cells.

### Cell Migration

The ability of cell migration is essential for cell growth, proliferation, the inputs and outputs of nutrients and signal intermediates, and normal cell physiology. Cell movement also enhances tumor cell invasion and metastasis (Svitkina, 2018). The process of cell migration is usually accompanied by changes in the actin cytoskeleton. Gα_12/13_ activates RhoA through their respective RhoGEF effectors and promotes the dynamic changes of cell shape controlled by actin cytoskeleton reorganization (Castillo-Kauil et al., 2020).

Gα_12/13_ plays pivotal roles in cell migration that occurs during development. For instance, the 5-hydroxytryptamine type 4 receptor (5-HT_4_R) triggers Gα_13_-mediated RhoA signal transduction, promoting the reorganization of filamentous actin and the morphology of mouse astrocytes, and enhancing hippocampal synaptic function (Müller et al., 2021). The 5-HT_4_R also mediates human endothelia cell migration and angiogenesis through the Gα_13_-RhoA-ROCK pathway *in vitro* (Profirovic et al., 2013). In zebrafish development, the S1PR2-Gα_13_-RhoGEF signal is necessary for the convergent movement of the endoderm by promoting myocardial migration at all stages of heart development (Ye et al., 2015). Disrupting the S1PR2-Gα_13_-RhoGEF pathway jeopardizes the endoderm’s convergence and the myocardium’s migration during the segmentation process (Ye and Lin, 2013). With the high concentrations of urotensin II stimulation, urotensin II receptors recruit Gα_13_ to activate the Rho-ROCK pathway and promote actin polymerization, which contributes to glioma cells invasion and new blood vessel formation (Lecointre et al., 2015). Gα_12_ participates in cell differentiation through the nuclear factor of activated T-cell c1 (NFATc1) and regulates cell migration and resorption through RhoA in the process of osteoclast formation in mice (Song et al., 2018). Resistance to inhibitors of cholinesterase 8A (Ric-8A) is a guanine nucleotide exchange factor of Gα subunits and an important partner of Gα_i_, Gα_q_, and Gα_13_ proteins (Papasergi-Scott et al., 2018). In *Xenopus* cranial neural crest cells, the Ric-8A-Gα_13_-focal adhesion kinase (FAK) signal regulates focal adhesion dynamics and neurite formation to control cell migration (Toro-Tapia et al., 2018). The activated Ric-8A catalyzes the nucleotide exchange on Gα_13_, induces the reorganization of the actin cytoskeleton, and promotes the migration of mouse embryonic fibroblasts (Wang et al., 2011).

Cell migration that is mediated by Gα_12/13_ is also important in metastasis. For example, the activated purinergic receptor 6 increases the number of filopodia and adhesions of human lung cancer cells (A549) and colorectal cancer cells (Caco-2) through both Gα_q_-Ca^2+^-PKCα and Gα_13_-ROCK signals, regulating cell migration (Girard et al., 2020). The GRPR-Gα_13_-RhoA-ROCK signaling is necessary for GRP to stimulate the migration of human colon cancer cells. This signaling also promotes the expression of COX-2, which contributes to cell migration (Patel et al., 2014). Similarly, Gα_12/13_ signal facilitates the invasion of human cervical cancer cells through a RhoA-ROCK-JNK signal axis (Yuan et al., 2016). Gα_12/13_ increases the gene expression of metalloproteinase-2 through p53, which is necessary for inducing the invasion and migration phenotypic changes of untransformed human breast epithelial cells (Kim et al., 2010). Gα_12_, which is overexpressed in many hepatocellular carcinoma patients, relieves the regulation of p53-responsive miRNA and promotes the metastasis and invasion of tumor cells (Yang et al., 2015). The Gα_12_-mediated pathway promotes metastasis and invasion of human nasopharyngeal carcinoma cells by regulating the reorganization of the actin cytoskeleton (Liu et al., 2009).

Increased Gα_12/13_ mediated cell migration also greatly contributes to primary solid tumor growth. Take as example that Gα_13_, which is up-regulated in breast cancer, inhibits the transcription of kallikreins through the RhoA-ROCK pathway and promotes the invasion and metastasis of breast cancer cells (Teo et al., 2016). The expression of *GNA13* in breast cancer cells is primarily regulated by MicroRNA (miR)-31, and the absence of miR-31 increases the expression of *GNA13* and the invasion of cancer cells (Rasheed et al., 2015). Similarly, Gα_13_ is overexpressed in pancreatic ductal adenocarcinoma and enhances the invasion of human pancreatic cancer cells in three-dimensional collagen by destroying cell adhesion; however, this process does not go through ROCK signal transduction (Chow et al., 2016). Gα_13_ is also involved in cell fusion (Carloni et al., 2013). Disintegrins and metalloproteinases stimulate Gα_13_ to activate RhoA. The up-regulated RhoA activity causes dephosphorylation of ezrin/radixin/moesin proteins and destruction of plasma membrane-cortical actin interaction, promoting fusion of human metastatic colon cancer cells and cell acquisition of drug resistance (Carloni et al., 2013).

These observations highlight the importance of Gα_12/13_-RhoA signaling in cell proliferation or migration. The expression of Gα_12/13_ and the downstream signaling are often elevated in cancer cells. However, in some disease states (especially cancer), the upstream ligand and receptor of Gα_12/13_ remain unknown.

### Immune Cell Function

Due to the lack of specific inhibitors of Gα_12/13_, the research of Gα_12/13_ in immune cells was stagnant for many years. However, with various transgenic models, the studies on the role of Gα_12/13_ in immune cells are accelerating.

#### T Cells

After naive T cells are activated, CD4^+^ T cells differentiate into different effector subsets: T helper (Th) 1, Th2, Th17, and T follicular helper (Tfh) cells, which perform their specific auxiliary functions (Dong, 2021). During T cell activation, Gα_12/13_ regulates actin polymerization and contributes to cell adhesion and migration (Wang et al., 2013). A recent study has found that Gα_13_-RhoA-ROCK2 signaling plays a key regulatory role in the differentiation and function of early Tfh cells. The Gα_13_-deficient Tfh cells impair the function of adhering B cells to form conjugates and stimulating B cells to produce immunoglobulins (Kuen et al., 2021). Morover, receptors coupled to Gα_12/13_ have been demonstrated to be essential for T cell adhesion, differentiation, and retention in lymph nodes (Moriyama et al., 2014; Mathew et al., 2019). For example, S1PR2 promotes the maturation of Tfh cells by directing co-localization with B cells in the germinal center; and the deficiency of S1PR2 in Tfh cells inhibits the retention in lymph nodes (Moriyama et al., 2014). In response to the stimulation of CXCL12, the Gα_13_-Rho signal in human T cells mediates the endosomal trafficking of CXCR4 (Kumar et al., 2011). CXCL12 also promotes the migration of Jurkat T cells through the CXCR4-Gα_13_-Rho signal axis (Tan et al., 2006). Meanwhile, Gα_12/13_ negatively regulates the activation state of integrin leukocyte-function-antigen-1 to modulate CD4^+^T cells trafficking and proliferation and susceptibility to immune diseases (Herroeder et al., 2009). In response to the activation of T cell receptor signals, the interleukin-2 inducible T cell kinase directly interacts with Gα_13_ to mediate the activation of SRF transcriptional activity (Huang et al., 2013). Conversely, Gα_12_ is a key mediator of T cell receptor-mediated interleukin-2 production and controls the differentiation of Th2 and Th17 cells (Won et al., 2010). Additionally, Gα_13_, but not Gα_12_, mediated signal transduction is necessary for early thymocyte proliferation and survival (McNeil Coffield et al., 2004).

#### B Cells

Mature B lymphocytes express LPAR2/5, LPA negatively regulates B cell receptor signaling through the LPAR5–Gα_12/13_–Arhgef1 pathway, inhibiting the release of calcium stored in the cell and the antibody response (Hu et al., 2014). Gα_12/13_ also regulates the maturation, migration, and polarization of marginal zone B cells. In mice with depletion of Gα_12/13_ in B cells, the number of zone B cells and zone B cell precursors are significantly reduced, but the formation of pseudopods is increased (Rieken et al., 2006).

Knockout of Gα_13_ also results in the loss of restriction of B cells in the germinal center, thus spreading to lymph nodes and blood (Muppidi et al., 2014). Meanwhile, Gα_13_ plays a direct role in the growth inhibition of B cells, affecting their survival and differentiation. It has been noted that the impaired phosphorylation of AKT at the Ser473 site is related to Gα_13_ activity and may affect the growth and survival of B cells (Green et al., 2011; O’Hayre et al., 2016). The mechanism by which the Gα_12/13_-RhoA axis inhibits the growth of B cells needs to be further explored.

#### Macrophages

In macrophages, complement C5a couples to Gα_12/13_ to activate Rho GTPases and tail retraction in migrating cells. Macrophages without Gα_12/13_ show complete chemotaxis but increased migration speed and a moderately impaired tail contraction (van den Bos et al., 2020). Thrombin, a major platelet activator, selectively induces the expression of CD36, a plasma membrane fatty acid transporter, and foam cell formation of RAW264.7 cells through PAR1-Gα_12_ signaling and facilitates atherosclerosis (Raghavan et al., 2018). Thrombin also induces the migration of monocytes or macrophages through the PAR1-Gα_12_ pathway (Gadepalli et al., 2013). The loss of Ric-8A in lymphocytes and bone marrow-derived macrophages leads to a decrease in the expression of Gα_i2/3_, Gα_q_, and Gα_13_, but not Gα_12_, leading to anemia and leukocytosis (Boularan et al., 2015).

The above studies suggest that dysregulation of Gα_12_ or Gα_13_ in immune cells may lead to pathophysiological consequences, such as cancer and autoimmunity. So far, the research of Gα_12/13_ in immune cells is relatively scarce. More research is needed to bring new therapeutic targets for cancers and autoimmune diseases.

### Gα_12/13_ Signaling and Diseases

Gα_12/13_ plays an important role in multiple stages of disease development in different tissues and organs. An increasing number of cancers have shown overexpressed Gα_12/13_, which is correlated to abnormal cell proliferation, metastasis, and invasion (Montgomery et al., 2014). The high abundance or constitutive activation of Gα_12_ and Gα_13_ are effective stimulators of oncogenic transformation. Gα_12/13_ also interacts with cell surface GPCRs and participates in the inflammation regulation (Dusaban et al., 2013; Hou et al., 2021). Meanwhile, Gα_12/13_ levels in metabolic organs, including liver and muscle, are altered in metabolic diseases (Koo et al., 2017; Kim et al., 2019). However, the mechanism by which Gα_12/13_ regulates the progression of these diseases has not been fully elucidated.

### Gα_12/13_ and Cancer

Gα_12/13_ are called the *gep* proto-oncogenes and are usually overexpressed in cancers (Yagi et al., 2016). Besides the functional roles in cancer cell migration and invasion, Gα_12/13_ and Rho GTPases exert pro- or anti-cancer effects in a cancer type and background-dependent manner.

The results of Gene Expression Omnibus and The Cancer Genome Atlas database analysis have shown that *GNA12* can be used as a biomarker for the personalized treatment of head and neck squamous cell carcinoma (HNSCC) patients and one of the prognostic genes for patients with HNSCC (Liu et al., 2021). *GNA13* is also a biomarker for the prognosis and metastasis of solid tumors, including HNSCC, ovarian cancer, lung cancer, and gastric cancer (Cerami et al., 2012; Gao et al., 2013; Cancer Genome Atlas, 2015). *GNA13* mutations are present at a high frequency in gastric cancer, nasopharyngeal cancer, prostate cancer, breast cancer, lymphoma, and bladder cancer (Muppidi et al., 2014; Wu et al., 2019; Arang and Gutkind, 2020).

The increased expression and activity of Gα_12/13_ contribute to the pathogenesis of different cancers. The copy number of *GNA12* is significantly increased in ovarian cancer, which enhances the function of Gα_12_ to promote cancer growth and metastasis (Wu et al., 2019). In two prostate cancer cell lines (C4-2B and PC3), both Gα_12_ and Gα_13_ are abundantly expressed (El-Haibi et al., 2013). The highly expressed *GNA13* also acts through Rho GTPase to drive the NF-κB transcription program and induce the expression of CXCL5 (Lim et al., 2019). A study on HNSCC has demonstrated that *GNA13* promotes the aggressive phenotype and drug resistance of tumor-initiating cells through the MAPK/AP-1 and NF-κB pathways (Rasheed et al., 2018). In bladder cancer, the Arg-200 (residue required to hydrolyze GTP) mutation of Gα_13_ strongly activates YAP/TAZ-dependent TEAD and myocardin-related transcription factor-A/B-dependent SRF transcriptional activities through the RhoGEF-Rho GTPase cascade (Maziarz et al., 2020). Additionally, the low expression of MiR-30b-5p in renal cell carcinoma up-regulates the activity of Gα_13_, which promotes cell proliferation, metastasis, and epithelial cell-mesenchymal transition (Liu et al., 2017). The reduction of regulators of G protein signaling protein (RGS) 12 promotes the Gα_12/13_-RhoA-YAP pathway and Ezrin expression, which leads to the growth and progression of osteosarcoma and lung metastasis (Li et al., 2021). Moreover, Gα_13_-mediated down-regulation of LATS1 promotes phenotypic changes of epithelial cell-mesenchymal transition in ovarian cancer cells (Yagi et al., 2019). These findings identify the Gα_13_-Hippo signaling as a potential target for cancer therapeutic interventions.

A few GPCR ligands have been identified upstream of Gα_12/13_ in cancers. LPA promotes the combination of Gα_12_ and EFA6 through LPAR2 and activates the ADP-ribosylation factors 6 mesenchymal pathway, thereby promoting the invasion, metastasis, and drug resistance of renal cancer cells (Hashimoto et al., 2016). High concentrations of LPA are also accumulated in the ascites of patients with ovarian cancer (Yu et al., 2016). The heterodimerization of LPAR1 and CD97 amplifies the LPA-mediated Gα_12/13_-Rho signal transduction and promotes the invasion of prostate cancer cells (Ward et al., 2011). Gastrin induces the activation of paxillin and FAK through the cholecystokinin B receptor-Gα_12/13_-RhoA-ROCK signaling pathway, thereby promoting the redirection of the Golgi apparatus and the directional migration of pancreatic cancer cells (Mu et al., 2018). Gastrin also promotes the activation of Gα_13_ through the gastrin type 2 cholecystokinin receptor in colon cancer cells. The recruitment of p190RhoGEF by Gα_13_ enhances the phosphorylation of FAK and paxillin, leading to RhoA activation and promoting the migration of cancer cells (Masià-Balagué et al., 2015). In addition, the down-regulated GPR65 in a variety of hematological malignancies promotes Gα_13_/Rho signal transduction, which leads to the decrease of c-myc oncogene expression and promotes growth, migration, and metastasis of blood cancer cells (Justus et al., 2017). Gα_13_ binds to CXCR5 in response to CXCL13 treatment and promotes prostate cancer cell movements; however, silencing Gα_13_ does not affect CXCL13-dependent cell invasion (El-Haibi et al., 2013).

Interestingly, the Gα_13_-Rho GTPase signaling has also been shown to alleviate hematological malignancies (Justus et al., 2017). In malignant tumors of the hematopoietic and lymphatic system, *GNA13* and RhoA mutations are present in B-cell lymphomas, mainly in DLBCL and Burkitt’s lymphoma (Justus et al., 2017). The mutation of *GNA13* in germinal center B cells is resistant to programmed cell death (Healy et al., 2016). These B cells are differentiated, facilitating genetic instability through continuous somatic hypermutation (Muppidi et al., 2014). Over time, the accumulation of driver mutations in persistent germinal center B cells may cause lymphoma. Loss of Gα_13_ facilitates germinal center B cells to spread to the lymph node and blood, leading to the pathogenesis of DLBCL (Healy et al., 2016). Moreover, in the absence of Gα_13_ signaling, S1P acts through S1PR3 to promote mouse germinal center B cells migration into the circulation (Muppidi et al., 2015). S1PR2 signals through Gα_13_ to induce tumor cell apoptosis and exert its tumor suppressor function (Flori et al., 2016). However, in DLBCL, the forkhead box protein 1 directly inhibits S1PR2 and promotes tumor cell survival (Flori et al., 2016). The restoration and activation of the Gα_13_-Rho pathway help reduce tumor growth and progression, supporting that the Gα_13_-RhoA axis has a tumor suppressor effect (Justus et al., 2017). Meanwhile, analyzing the genome of tumor tissues from patients with classical Hodgkin lymphoma has revealed that *GNA13* is one of the genes repeatedly mutated (Tiacci et al., 2018). *GNA13* variants are mostly heterozygous, including nonsense, frameshift, and missense mutations, like the mutation patterns in DLBCL and Burkitt’s lymphoma (Muppidi et al., 2014; Justus et al., 2017).

Despite the emerging evidence supporting the critical roles of Gα_12/13_ in cancers, many questions remain about how the ligands and receptors trigger Gα_12_ or Gα_13_ signaling in a cancer type-specific manner. A comprehensive understanding of these mechanisms may provide a promising prospect for targeting specific Gα_12/13_ signaling processes in cancer therapies.

### Gα_12/13_ and Inflammation

Gα_12/13_ has been implicated in both induction and suppression of inflammation. In the context of inflammation, thrombin, LPA, and S1P transmit signals through Gα_12/13_-coupled receptors to amplify inflammatory responses (Gavard and Gutkind, 2008; Dusaban et al., 2017; Lee et al., 2019).

Early studies have found a direct connection between Gα_12/13_ signaling and arachidonic acid in cells (Dermott et al., 1999; Mariggiò et al., 2006). NIH-3T3 cells transformed with Gα_12_QL show increased secretion of arachidonic acid and transcriptional activation of cyclooxygenase-2 (Dermott et al., 1999). Thrombin stimulates CHO cells to activate the Gα_13_-RhoA-ERK1/2 signaling through PAR1, leading to the phosphorylation of cytosolic phospholipase A2 and the increase of arachidonic acid (Mariggiò et al., 2006). The activation of LPAR and S1PR also promotes the expression of inflammatory genes (such as cyclooxygenase-2) and the proliferation of astrocytes through Gα_12/13_-RhoA-PLCɛ-protein kinase D (PKD)-NF-κB signaling, which contributes to neuroinflammation (Dusaban et al., 2013).

Recent work has shown that S1P promotes the NLRP3 inflammasome-mediated inflammatory response and the secretion of pro-inflammatory cytokines such as interleukin-1β and interleukin-18 through the S1PR2-Gα_12/13_-MAPK pathway (Hou et al., 2021). Meanwhile, a variety of pro-inflammatory ligands promote the interaction of Gα_13_ to VE-cadherin, inducing Src activation and VE-cadherin phosphorylation, leading to the internalization of VE-cadherin, the loss of endothelial barrier function, and vascular inflammation (Gong et al., 2014). The deficiency of Gα_13_ in leukocytes and platelets inhibits thrombosis and inflammation and significantly optimizes the survival rate of septic mice (Cheng et al., 2021).

Gα_12_ and Gα_13_ have opposite effects on osteoclasts. The silencing of Gα_13_ enhances the AKT-GSK3β-NFATc1 signal cascade by inhibiting RhoA, strongly promoting the formation and size of osteoclasts (Wu et al., 2017). This observation depicts that Gα_13_ is the main endogenous negative switch for osteoclast production. Gα_13_ also protects against various bone loss disease models from inflammation (Wu et al., 2017; Nakano et al., 2019). In comparison, a study has shown Gα_12_ is involved in the pathophysiology of bone diseases such as osteoporosis or rheumatoid arthritis (Song et al., 2018). Interestingly, the volume of trabecular bone increases in Gα_12_ knockout mice, whereas the number of osteoclasts decreases, contrary to the phenotype of Gα_13_ deletion (Song et al., 2018). The underlying mechanisms of these opposite phenotypes are unclear, possibly because that Gα_12_^-/-^ mice are whole body knocked out, whereas Gα_13_^-/-^ mice undergo an osteoclast lineage-specific conditional knockout.

### Gα_12/13_ and Metabolic Diseases

Gα_12_ is extensively expressed in metabolic organs, for instance, the liver (Strathmann and Simon, 1991; Kim et al., 2018a). Fasting of normal mice for 24–48 h significantly enhances the expression of Gα_12_ in the liver (Kim et al., 2018a). *GNA12*-knockout mice are prone to hepatic steatosis and obesity after being fed a high-fat diet due to reduced energy consumption (Kim et al., 2018a). A study using cDNA microarray analysis with the liver of *GNA12*-knockout mice has found that Gα_12_ regulates mitochondrial respiration through the Sirtuin 1/PPARα network (Kim et al., 2018a). Sirtuin 1, a class III histone deacetylases activated by NAD^+^, is an important regulator involved in the fatty acid oxidation transcription network (Kalliora et al., 2019). Gα_12_ induces ubiquitin-specific peptidase 22 through HIF-1α to promote the stability of Sirtuin 1, controlling lipid metabolism and mitochondrial respiration (Kim et al., 2018a). Interestingly, Gα_12_ also exists in the endoplasmic reticulum and participates in the endoplasmic reticulum export (Subramanian et al., 2019). With the coat protein II subunit Sec24 binds to the cargo, it acts as a GEF to activate Gα_12_ at the export site of the endoplasmic reticulum, promoting cargo export and inhibiting protein synthesis (Subramanian et al., 2019).

The role of Gα_13_ in energy metabolism has opposite effects in skeletal muscle (prodiabetic) and liver (antidiabetic) (Koo et al., 2017; Kim et al., 2019). Gα_13_ is more highly expressed in skeletal muscle than other metabolic organs (Koo et al., 2017). The knockout Gα_13_ in skeletal muscles contributes to systemic energy homeostasis, increasing glucose metabolism and insulin sensitivity and inhibiting diet-induced obesity and hepatic steatosis (Koo et al., 2017). The level of Gα_13_ in skeletal muscle is reduced by exercise but is increased under conditions of metabolic diseases. Loss of Gα_13_ in skeletal muscle inhibits the RhoA-ROCK2 pathway and activates NFATc1, which induces the conversion of skeletal muscle fibers into oxidized form and enhances energy metabolism (Koo et al., 2017). On the contrary, the lack of Gα_13_ in the liver of mice leads to the overproduction of inter-α-trypsin inhibitor heavy chain 1, which exacerbates systemic insulin resistance (Kim et al., 2019).

GPR40 is a clinically proven molecular target for the treatment of diabetes. A GPR40 allosteric full agonist promotes glucose-stimulated insulin secretion in pancreatic β cells through GPR40-mediated Gα_12_ signaling (Rives et al., 2018). In response to mechanical overload, GPR56 elevates the expression of the mammalian target of rapamycin and insulin-like growth factor 1 through Gα_12/13_ and promotes muscle protein synthesis and myotube hypertrophy (White et al., 2014). Succinate activates the Gα_12_-PKC-p38-dynamin-related protein 1 signaling through GPR91 to increase mitochondrial fission, enhances ATP production and membrane potential of mitochondria, and promote the migration of human mesenchymal stem cells (Ko et al., 2017). LPA is a key product of fatty acid metabolism. LPAR4 is selectively coupled with Gα_12/13_ in adipocytes to limit the remodeling and healthy expansion of white adipose tissue in high-fat diet mice (Yanagida et al., 2018). The above studies have shown that the GPCR-Gα_12/13_ axis can be an attractive target for treating metabolic diseases.

### Gα_12/13_ and Fibrotic Diseases

Gα_12/13_ has a pro-fibrotic effect mainly mediated by the RhoA-ROCK activation (Haak et al., 2020). Early studies have demonstrated that Gα_12/13_ play a key role in regulating the phenotype of cardiac fibroblasts (Fujii et al., 2005; Nishida et al., 2007). The stimulation of Ang II induces the production of reactive oxygen species (ROS) through the AT_1_R-Gα_12/13_-Rac pathway. The ROS-mediated JNK activation promotes the activity of AP-1 and NFAT, leading to the proliferation of cardiac fibroblasts (Fujii et al., 2005). Recently, it has been found that targeted inhibition of the Gα_12/13_-RhoA-ROCK pathway successfully alleviates Ang II-induced cardiac dysfunction and the fibrotic response of cardiac fibroblasts (He et al., 2017). Endothelin-1 activates the Endothelin-1 type A receptor-Gα_12/13_ axis in cardiac fibroblasts and promotes the formation of myofibroblasts through Rac-dependent ROS production and JNK activation (Nishida et al., 2007). The osteoglycin in the heart binds to LPAR3 and mediates the Gα_12/13_-Rho-ROCK signal to attenuate the transactivation of epidermal growth factor receptors, thus inhibiting the proliferation and migration of cardiac myofibroblasts and negatively regulating cardiac fibrotic remodeling (Zuo et al., 2018).

Among numerous G protein members, Gα_12_ is expressed abundantly in hepatic stellate cells of the fibrotic liver, whereas Gα_13_ is not (Kim et al., 2018c). The imbalance of miR-16 in hepatic stellate cells leads to the overexpression of Gα_12_, which promotes autophagy through the transcription of JNK-dependent autophagy-related genes 12-5 and accelerates the progression of liver fibrosis (Kim et al., 2018c). Meanwhile, deficiency of Gα_12_ significantly blocks bleomycin-induced pulmonary fibrosis in mice. LPA stimulates the phosphorylation and activation of PKC-δ through Gα_12_ and the mammalian target of rapamycin complex 2, which is important for fibroblast migration and the development of pulmonary fibrosis (Gan et al., 2012).

In short, the role of Gα_12_ or Gα_13_ in fibrotic diseases is unclear. Using various omics (e.g., genomics, proteomics) to detect the expression of Gα_12/13_ in specific tissues/cells of fibrotic diseases, exploring its upstream receptors and downstream signals may lead to a better understanding of the pathological mechanisms of the fibrotic diseases.

### Gα_12/13_ and Circulatory and Renal Disorders

Gα_13_ is involved in heart remodeling induced by pressure overload and plays a central role in the transition to heart failure (Takefuji et al., 2012). The Ang II-AT_1_R-Gα_13_-RhoA-myocardin-related transcription factor signal cascade regulates the expression of hypertrophy and fibrosis genes in cardiomyocytes (Takefuji et al., 2012). In response to low concentrations of thrombin, disabled-2, a linker protein, regulates Gα_12/13_-mediated RhoA-ROCK activation and enhances ADP release, which increases the activity of AKT and mammalian target of rapamycin and promotes platelet aggregation and thrombosis (Tsai et al., 2014). Thrombin also stimulates platelets to promote the interaction of Gα_13_ and receptor-interacting protein kinase 3. Receptor-interacting protein kinase 3 participates in the integrin-Gα_13_ signal to promote platelet activation and thrombosis (Zhang et al., 2017). The newly developed high-load ExE peptide nanoparticles, based on the Gα_13_ binding ExE motif on the cytoplasmic domain of integrin β_3_, inhibit thrombosis and protect mice from cardiac I/R injury (Pang et al., 2020). This study supports that the integrin-Gα_13_-RhoA-YAP pathway can be targeted for anti-atherosclerosis therapy (Wang et al., 2016). Inhibiting proteins that specifically interact with Gα_13_ is a promising approach for the treatment of diseases such as macular degeneration, atherosclerosis, and tumor angiogenesis (Wang et al., 2017). In the process of angiogenesis, RGS5 converts Gα_q/11_-mediated calcium-dependent contraction to Gα_12/13_-mediated RhoA activation, leading to the formation of stress fibers in the vascular smooth muscle cells of the artery and the process of vascular remodeling (Arnold et al., 2014). Gα_12/13_ are also essential for blood vessel formation. Gα_13_ interacts with Abl1 to form a complex, which regulates actin cytoskeleton reorganization, remodeling, and endothelial cell migration and promotes blood vessel formation (Wang et al., 2017).

Gα_12_ also plays a key role in inducing renal I/R injury through various mechanisms, such as destruction of tight cell connections, oxidative stress, inflammation, and cell apoptosis. Gα_12_ knockout mice are almost completely protected from I/R injury (Yu et al., 2012). In addition, kidney injury molecule-1 prevents tissue damage by blocking the binding of GTP to Gα_12_ in renal I/R (Ismail et al., 2015). Gα_12_ is also necessary for the development of a mouse renal cyst model induced by polycystin-1 mutation. The lack of polycystin-1 promotes the activation of Gα_12_, leading to alteration in the form of N-cadherin, destroying cell-matrix/cell adhesion, inhibiting FAK, but promoting the formation of stress fibers (Wu et al., 2016). The activation of Gα_12_ in mouse podocytes also leads to the imbalance of collagen expression in glomeruli, age-dependent proteinuria, and focal glomerular sclerosis (Boucher et al., 2012). However, a study has found that the activation of the dopamine D3 receptor increases coupling to Gα_12_, leading to the reduction in ROS production and inflammation, ultimately preventing renal I/R injury (Wang et al., 2015).

The coupling of different GPCRs to Gα_12/13_ and the impacts on the pathophysiology have not been fully elucidated. The identification of the reciprocal binding domains of Gα_12/13_ to related cellular proteins will facilitate the development of more specific inhibitors to selectively disrupt the interaction. A comprehensive understanding of the complex regulatory mechanisms of Gα_12/13_ signaling may lead to novel therapeutics targeting specific functions of Gα_12_ or Gα_13_ in a disease-specific manner.

## Conclusion

The mechanisms of signal transduction through Gα_12/13_ are still one of the most enchanting problems in biology. Many crucial questions remain to be addressed: what determines the specificity of the receptor-Gα_12/13_ interaction (Montgomery et al., 2014; Lymperopoulos et al., 2021)? What determines the specific selectivity of the receptor to Gα_12_ or Gα_13_ (Corbisier et al., 2015; Mackenzie et al., 2019)? Do receptor and Gα_12/13_ form a pre-coupling complex (Ayoub et al., 2012; Zhang et al., 2017)? The importance of Gα_12/13_ in mediating the complexity of signals, affecting the diversity of cell functions, and participating in the pathogenesis of diseases are continuously being explored. Gα_12/13_ are pathologically relevant to cancer, bone diseases, liver steatosis, and pulmonary fibrosis. Gα_12/13_ are considered an attractive biomarker and target for diagnosing and treating related diseases. However, it is unwise to directly stimulate or inhibit the activity of Gα_12/13_ due to the complexity of its regulatory network, the diversity of functions, and the high likelihood of off-target effects. In comparison, stimulating/inhibiting the upstream receptor/downstream pathways of Gα_12_ or Gα_13_ in specific organs/cells may offer a chance of successful therapy. A better understanding of the ligand-receptor-Gα_12/13_-downstream signaling networks may guide new drug development in the future.

## Glossary

5-HT4R: 5-hydroxytryptamine type 4 receptor
AKT: protein kinase B
Ang II: angiotensin II
AP-1: activator protein 1
AT1R: Ang II type 1 receptor
CXCL12: C-X-C motif chemokine 12
CXCR4: C-X-C chemokine receptor 4
DLBCL: diffuse large B-cell lymphoma
ERK: extracellular regulated protein kinases
FAK: focal adhesion kinase
FZD: Frizzled
GEF: guanine nucleotide exchange factors
GPCRs: G protein-coupled receptors
GRP: gastrin-releasing peptide
GPR56: g protein-coupled receptor 56
HNSCC: head and neck squamous cell carcinoma
I/R: ischemia/reperfusion
JNK: c-Jun N-terminal kinase
LATS1/2: large tumor suppressor 1/2
LPA: lysophosphatidic acid
LPAR: LPA receptor
MAPK: mitogen-activated protein kinase
MRTF: myocardin-related transcription factor
NFAT: activated T cell nuclear factor
NFATc1: nuclear factor of activated T-cell c1
NF-κB: nuclear factor-kappa B
PARs: protease-activated receptors
PI3K: phosphatidylinositol 3-kinase
PKC: protein kinase C
PLC: phospholipase C
PPARγ: peroxisome proliferator-activated receptor-γ
RGS: regulators of G protein signaling
RhoGEF: Rho guanine nucleotide exchange factor
RhoA: ras homolog family member A
Ric-8A: resistance to inhibitors of cholinesterase 8A
ROCK: Rho-associated protein kinase
ROS: reactive oxygen species
S1P: sphingosine 1-phosphate
SRF: serum response factor
Tfh: T follicular helper
Th: T helper
WNT: Wingless/Int-1 lipoglycoprotein
YAP/TAZ: yes-associated protein/transcriptional coactivator with PDZ-binding motif
